# Sex differences in beneficial and pathogenic bacteria in People With HIV (PWH) with a history of heavy alcohol drinking

**DOI:** 10.3389/fmicb.2025.1632949

**Published:** 2025-11-21

**Authors:** Aakarsha V. Rao, Smita S. Ghare, Vasuk Gautam, Kristi L. Hoffman, Joseph Petrosino, Kaku So-Armah, Jeffrey H. Samet, Gregory J. Patts, Debbie M. Cheng, Elena Blokhina, Evgeny M. Krupitsky, Dmitry Lioznov, Edwin Zvartau, Craig J. McClain, Hillary Tindle, Matthew S. Freiberg, Shirish S. Barve

**Affiliations:** 1 Department of Medicine, University of Louisville, Louisville, KY, United States; 2 Alcohol Research Center, University of Louisville, Louisville, KY, United States; 3 Neuroscience Department, Norton Research Institute, Louisville, KY, United States; 4 Department of Molecular Virology and Microbiology, Baylor College of Medicine, Houston, TX, United States; 5 Center for Metagenomics and Microbiome Research, Baylor College of Medicine, Houston, TX, United States; 6 Boston University School of Medicine, Boston, MA, United States; 7 Center for Health Data Science (CHDS), Boston University School of Public Health, Boston, MA, United States; 8 Department of Biostatistics, Boston University School of Public Health, Boston, MA, United States; 9 Department of Addictions, Bekhterev National Medical Research Center for Psychiatry and Neurology, St. Petersburg, Russia; 10 Valdman Institute of Pharmacology, First Pavlov State Medical University, St. Petersburg, Russia; 11 Robley Rex VA Medical Center, University of Louisville, Louisville, KY, United States; 12 Department of Medicine, Vanderbilt University School of Medicine, Nashville, TN, United States; 13 Geriatric Research Education and Clinical Center (GRECC), Veterans Administration Tennessee Valley Healthcare System, Nashville, TN, United States

**Keywords:** gut dysbiosis, HIV, alcohol, sex, butyrate, 16S rRNA gene sequencing

## Abstract

**Background:**

HIV-1 infection and hazardous levels of alcohol consumption have been independently linked to gut dysbiosis affecting beneficial butyrate-producing bacteria. However, sex-based differences in the composition and function of gut microbiome of People With HIV (PWH) with a history of heavy alcohol drinking remain undetermined, which is the focus of this study.

**Methods:**

Cross-sectional study examining structural and functional features of the gut microbiome in PWH between men and women with a history of hazardous alcohol drinking recruited at St. Petersburg, Russia. 16S rDNA sequencing information was used for metataxonomic, Phylogenetic Investigation of Communities by Reconstruction of Unobserved States (PICRUSt2) and Linear Discriminant Analysis Effect Size (LEfSe) analyses. Group-wise comparisons were done using Mann–Whitney *U*-test. Further, linear and logistic regression models were used to evaluate the association between sex and measures of gut microbial dysbiosis and Firmicutes/Bacteroidota (F/B) ratio, respectively. Data were adjusted for confounding covariates particularly, HIV-viral load, Anti-retroviral Therapy (ART) and alcohol usage.

**Results:**

Metataxonomic analysis demonstrated that women depicted significantly higher microbial diversity (Operational Taxonomic Units, OTUs and Shannon Index), higher percent relative abundance (%RA) of *Firmicutes*, lower %RA of *Bacteroidota* and higher F/B ratio. Importantly, logistic regression revealed that women had twice the odds of having F/B ratio > 1. Notably, women demonstrated significantly higher %RA of butyrate-producing bacterial families, i.e., *Lachnospiraceae, Oscillospiraceae, Rikenellaceae* and *Marinifilaceae* and genera. Correspondingly, significantly greater expression of bacterial genes involved in butyrate synthesis in women was demonstrated by PICRUSt2 analysis. Additionally, women depicted lower %RA of pathobiont, *Prevotellaceae* particularly, *Prevotella_9* genus.

**Conclusion:**

Overall, we observed significant sex-based differences in the relative abundances of beneficial bacterial communities such as butyrate producers and potential pathogenic Prevotella community in the gut microbiome of PWH with a history of heavy alcohol consumption. The observed sex-based differences are clinically relevant and could inform therapeutic strategies with evidence-based probiotics.

## Introduction

Hazardous alcohol drinking is common among people with HIV (PWH) (Duko et al., 2019; Williams et al., 2016; Molina et al., 2014). Pathological changes as a result of both HIV-1 infection and heavy alcohol drinking share several salient features. Particularly, heavy alcohol drinking and HIV-1 infection are known to be independently associated with alterations in the structural and functional features of the gut microbiome leading to dysbiosis (Yan et al., 2021; Chen et al., 2011; Dillon et al., 2017). In the context of HIV-1 infection alone, gut dysbiosis is characterized by a total decrease in microbial diversity due to a decrease in beneficial bacteria and an increase in opportunistic pathogens (Dillon et al., 2017; McHardy et al., 2013). Additionally, a clinical study published by our group highlights changes in the gut microbiome composition driven by loss of beneficial butyrate-producing bacterial communities associated with age as a consequence of HIV-1 infection alone in older participants with HIV between the ages of 50–70 years (Ghare et al., 2022). Similar to HIV infection, heavy alcohol drinking is also associated with shifts in the gut microbiome composition (Hartmann et al., 2015; Day and Kumamoto, 2022; Mutlu et al., 2012). Further, both pre-clinical and clinical work have observed alcohol-mediated reduction in the relative abundances of beneficial butyrate-producing bacterial communities (Singhal et al., 2021; Litwinowicz and Gamian, 2023). However, the combinatorial effects of both hazardous alcohol drinking and HIV-1 infection in the same population remain largely undetermined.

Earlier work has estimated combined prevalence of Alcohol Use Disorder (AUD) among PWH is 29.8%, with higher occurrence in men than women (Duko et al., 2019). Furthermore, it has been well established that sex is a significant determinant of differences in the structural features of the gut microbiome in healthy individuals (Kim et al., 2020; Mueller et al., 2006; Ding and Schloss, 2014; Koliada et al., 2021; Takagi et al., 2019; Yoon and Kim, 2021). In the context of HIV infection, sex-based differences in the gut microbiome are only beginning to be identified. However, in men with HIV infection, sexual behavior seems to be an important determinant of gut microbial dysbiosis. In this regard, gut microbial dysbiosis marked by a significant enrichment in Prevotella is observed in men who have sex with men (MSM) compared to non-MSM individuals (Vujkovic-Cvijin et al., 2020; Rocafort et al., 2024). Further, with regards to heavy alcohol consumption, pre-clinical studies demonstrate sex-based differences in the gut microbiome between male and female mice subjected to chronic and chronic-binge ethanol exposure models (Domínguez-Pino et al., 2024; Pacheco et al., 2025). However, clinical studies demonstrating sex-based differences in the gut microbiome between men and women with AUD are limited (Koutromanos et al., 2024, 2025; Grodin et al., 2024). Moreover, the combinatorial effects of HIV-1 infection and heavy alcohol consumption on the differences in the gut microbiome between men and women have not been examined. In view of this, studying the interactive effects of heavy alcohol drinking and HIV-1 infection on the gut microbiome, with a focus on sex-dependent differences, is clinically relevant for understanding disease progression in men and women and developing effective, targeted therapies for PWH. To obtain specific insights in the potential sex-based differences, the current study examined the structural and functional features of the gut microbiome in PWH with a history of heavy alcohol consumption between men who are predmoninantly reported to have sex with women (MSW) and women. We pursued a cross-sectional analysis of 202 PWH with a history of heavy alcohol drinking (men = 143, women = 59) from St. Petersburg, Russia. Specifically, we profiled the fecal microbiome of these study participants employing 16S rRNA gene sequencing along with predictive analysis of functional characteristics of the gut microbiome using PICRUSt2. Our analysis unraveled critical sex-based differences in gut microbiome composition with a particular focus on beneficial butyrate-producing bacteria and inflammophilic pathogenic Prevotella bacteria. Additionally, this study demonstrates the sex-based differences in predicted functional characteristics of butyrate-producing bacteria in expressing genes involved in the four major butyrate-producing pathways in humans.

## Materials and methods

### Study design and participants

Study participants (*N* = 202) were recruited from the “The Studying Partial-agonists for Ethanol and Tobacco Elimination in Russians with HIV (St PETER HIV)” trial. This was a four-arm randomized, double-blinded, placebo-controlled, clinical trial among 400 PWH who are daily smokers with risky alcohol use recruited from HIV clinical care sites in St. Petersburg, Russia between July 2017 and December 2019. All participants were between 24 and 67 years old; HIV-positive; experienced ≥ five heavy drinking days [HDD] defined by the National Institute on Alcohol Abuse and Alcoholism [NIAAA] at-risk drinking levels (risky drinking) (Alcoholism NIoAAa, 2024a) in the past 30 days; smoked an average of 21.3 cigarettes per day in the past 30 days, and had a willingness to reduce alcohol, smoking or both. Exclusion criteria included lack of fluency in Russia, pregnancy or planning to become pregnant; breastfeeding; took smoking cessation medications in the past 30 days; had cognitive impairment resulting in an inability to provide informed consent; unstable psychiatric illness; history of seizures; or known allergy to study medications. This study was approved by Institutional Review Boards at Boston University Medical Campus (IRB# H-3668), Vanderbilt University Medical Center (IRB# 171584), and First Pavlov State Medical University (IRB# Case 32/17-H). Participant safety data was reviewed by a Data Safety and Monitoring Board every 6 months. All study participants provided written informed consent.

### Participant assessments and biologic specimen collection

Participants completed baseline questionnaires from the St PETER HIV trial which included the following items: demographics, 30-day alcohol and cigarette timeline follow back (TLFB), Alcohol Use Disorder Identification Test (AUDIT), anti-retroviral therapy (ART) use and adherence, and other substance use. Additionally, a modified food frequency questionnaire, and survey items focused on recent antibiotic use and probiotic use. Participants also provided blood samples for CD4 cell count, HIV RNA, and Phosphatidylethanol (PEth) levels, biomarker of alcohol consumption. The participants in this study provided a fecal sample within 30 days of baseline examination, and had no use of antibiotics, probiotics, or prebiotics in 3 months prior to baseline examination.

### Alcohol assessment

Alcohol consumption patterns were assessed by total grams of alcohol in the past 30 days, which was converted to number of drinks per week using the following formula (there is 14 grams of alcohol in a standard drink; Alcoholism NIoAAa, 2024b):


$$\text{Drinks}\;\mathrm{per}\;\text{week}=\text{grams of alcohol}\;\mathrm{per}\;\text{month}\;x\;\frac{1}{14}\;x\;\frac{7}{30}$$

$$\text{Drinks}\;\mathrm{per}\;\text{week}=\text{grams of alcohol}\;\mathrm{per}\;\text{month}\;x\;\frac{1}{60}$$

### Gut microbiome assessment

Fecal samples were used to perform 16S rRNA gene sequencing. The sequencing method was adapted from the NIH-Human Microbiome Project and the Earth Microbiome Project (Human Microbiome Project Consortium, 2012a; Human Microbiome Project Consortium, 2012b; Thompson et al., 2017). Briefly, total genomic DNA is extracted using the Qiagen MagAttract PowerSoil DNA Kit (Qiagen, Redwood City, CA). The 16S rDNA V4 region was amplified by PCR and sequenced on the MiSeq platform (Illumina, San Diego, CA; RRID: SCR_016379) using the 2×250 bp paired-end protocol, yielding paired-end reads that overlap almost completely (Caporaso et al., 2012). Resulting reads are denoised using the Deblur algorithm (Amir et al., 2017) following the default workflow and the length limit set to 252 bp. The generated representative sequences are mapped against an optimized version of the latest current SILVA Database (Quast et al., 2013) (RRID: SCR_006423) containing only sequences from the v4 region of the 16S rRNA gene to determine taxonomies using usearch70 ‘usearch_global’ function (Edgar, 2010), specifying the identity threshold to 97%. Phylogeny information contained in the biom file is generated by aligning the centroid sequences with MAFFT (Katoh and Toh, 2010) (RRID: SCR_011811) and creating a tree via FastTree (Price et al., 2010) (RRID: SCR_015501). The biom file was summarized, recording the number of reads per sample, and merged with a file to produce a final summary file with read statistics and taxonomy information.

### Inferred metagenomics (PICRUSt2 analysis)

Phylogenetic Investigation of Communities by Reconstruction of Unobserved States (PICRUSt2; RRID: SCR_022647) was used for predictive analysis of butyrate synthesizing pathways in the gut microbiome of study participants in this cohort (Douglas et al., 2020). Default settings of the q2-PICRUSt2 plugin, which utilizes sequence and gene data information generated in the form of QIIME2 (Quantitative Insights Into Microbial Ecology) output (Bolyen et al., 2019) with Deblur denoising algorithm. The 16S rRNA gene data were used along with information pertaining to the copy numbers of butyrate synthesizing genes present within each sequenced archaeal and bacterial taxonomic group in the Integrated Microbial Genomes (IMG) database. The sequence placement with the reference genome database was completed using the SEPP (SATé-enabled phylogenetic placement) pipeline (Mirarab et al., 2012) of the q2-PICRUSt2 plugin. The output file contains normalized values corresponding to the contribution of bacterial genome in a particular sample toward expression of a specific Kyoto Encyclopedia of Genes and Genomes ID (KEGG Ortholog ID; RRID: SCR_012773) (Douglas et al., 2020).

### Statistical analyses

Descriptive statistics were used to characterize the demographic and clinical characteristics of the study population. Sex-based differences in gut microbiome composition were evaluated using Mann–Whitney *U*-tests. These statistical tests were performed using GraphPad Prism, version 10.1.1 (270). Separate multiple linear regression models were created to evaluate unadjusted and adjusted association between sex and measures of gut microbial composition and dysbiosis such as, alpha diversity metrics (Operational Taxonomic Units, OTUs and Shannon Index), F/B ratio, percent relative abundances (%RA) of total butyrate-producing genera and pathobiont family *Prevotellaceae*. Adjusted models included covariates: age, log10 of HIV viral load, CD4 count, opioid use and drinks per week in the last 30 days. SAS statistical software version 9.4 was used in the analysis. Logistic regression analysis was performed in Python version 3.11.5 using the statsmodel.api library (Seabold SaJP, 2010) to evaluate the effect of sex on F/B ratio. Odds ratio measure was calculated by exponentiating the regression coefficient obtained in the model; the confidence intervals were obtained by exponentiating the lower and upper bounds of confidence interval for the regression coefficient. SciKit learn library in Python (Fabian Pedregosa et al., 2011) was used to evaluate the predictive accuracy of the model.

## Results

### Clinical characteristics and demographics of people with HIV (PWH) with a history of heavy alcohol drinking

This cross-sectional study involved 202 PWH with a history of heavy alcohol drinking, whose median age was 38 years (mean ± SD = 38.8 ± 6.3 years). The main characteristics of the population are enlisted in Table 1. Of the 202 study participants, 143 were male (71%) and 59 were female (29%) with no documented use of antibiotic, prebiotic or probiotic 3 months prior to enrollment. With regards to sexual behavior, only 12.59% men reported to have sex with men (MSM) and 5.08% women reported to have sex with women (WSW). Further, 142 participants (70.3%) were ART compliant. Amount of alcohol consumed by participants was measured as number of drinks per week ranging from 4.9 to 108.9 over the last 30 days.

**Table 1:** Demography and clinical characteristics of study subjects: summary of study cohort metadata including age, sex, CD4 T-cell counts, HIV viral load, current ART usage, amount of alcohol consumed and sexual behavior.

Demographics	Median (IQR)		Mean ± SD		
	Male	Female	Male	Female	Significance (*p*-value)
No. of samples	143	59	143	59	
Age (years)	39 (35–43)	37 (35–41)	39.1 ± 6.2	38.2 ± 6.4	0.356
CD4 T-cell count	361 (209–549)	345 (194–501)	391.4 ± 240.1	374.1 ± 256.2	0.648
HIV viral load (log10)	2.176 (2.176–4.446)	2.176 (2.176–4.684)	3.3 ± 1.35	3.27 ± 1.52	0.69
Is the participant on ART medication? Yes (%)	NA	NA	101 (70.6%)	41 (69.5%)	0.872
Total grams of alcohol (30 days)	924.4 (597.1–1,411)	647.2 (476–980)	1,203 ± 942.6	903.7 ± 808.3	0.0007
Total number of drinks per week (30 days)	15.41 (9.95–23.52)	10.79 (7.93–16.33)	20.0 ± 15.7	15.1 ± 13.5	0.034
Sexual behavior (Men who have sex with Women, MSW)	NA	NA	123 (86.01%)	55 (93.22%)	

Data shown as mean ± SD. Mann–Whitney *U*-test was performed to evaluate sex-based differences for all the clinical characteristics.

### Sex-based differences in gut microbial diversity in PWH with a history of heavy alcohol drinking

**Figure 1:**
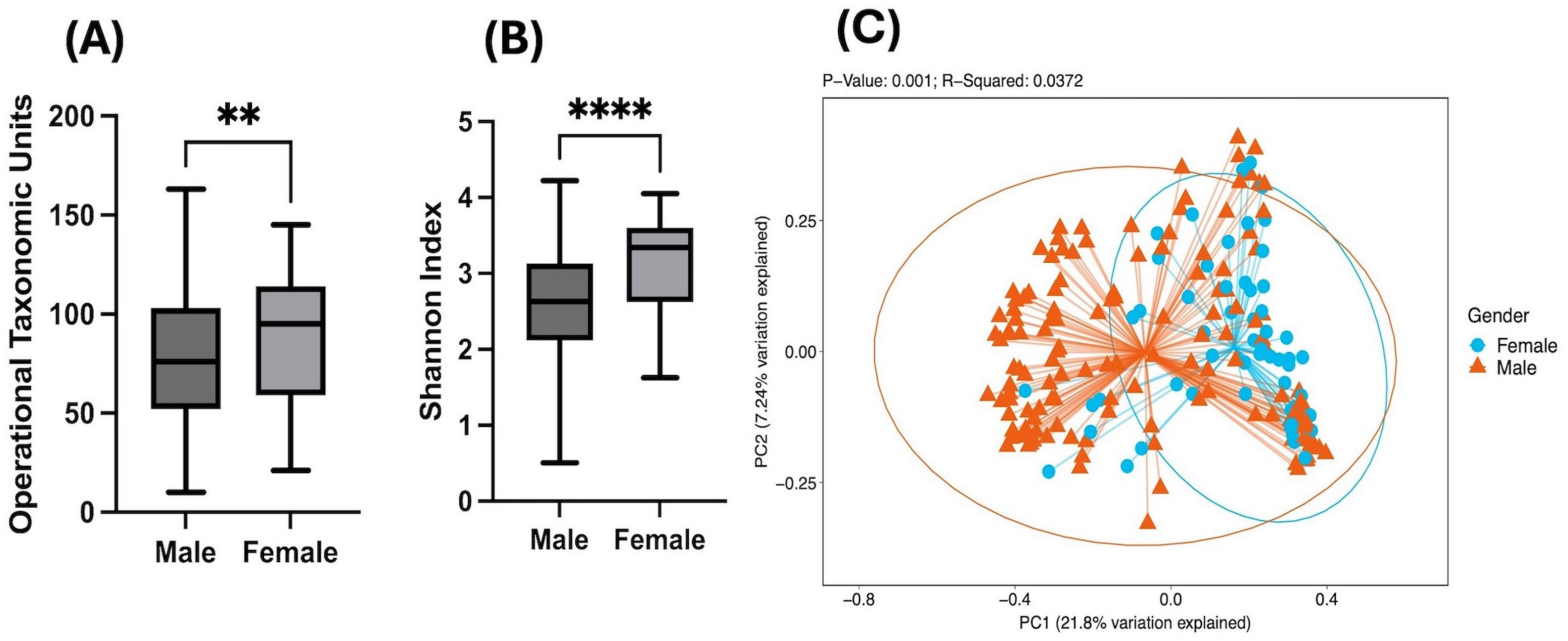
Sex-based differences in gut microbial diversity: Bar graphs showing unadjusted Mann–Whitney *U*-test comparison in PWH between men and women having a history of heavy alcohol drinking for alpha diversity metrics **(A)** OTUS and **(B)** Shannon Index. Statistical analysis shown as **p < 0.01; ***p < 0.001; ****p < 0.0001; **(C)** PCoA plot depicting unadjusted Weighted Bray-Curtis Beta Diversity analysis showing sex-based clustering of the microbiome composition.

To evaluate the combinatorial effects of heavy alcohol drinking and HIV-1 infection on the compositional drifts in the gut microbiome, fecal samples of study participants were processed to sequence the bacterial 16S rRNA marker gene. Gut microbiome diversity was assessed by alpha diversity indices, OTUs and Shannon Index. Comparisons between men and women without adjusting for covariates showed that women demonstrated significantly greater OTUs (microbial mass; *p* < 0.01; Figure 1A) and Shannon Index (evenness; *p* < 0.0001; Figure 1B). These sex-based differences in gut microbial diversity continued to remain statistically significant even when adjusted for covariates such as age, log10 of HIV viral load, current ART usage, CD4 count, opioid use and number of drinks consumed per week over 30 days (Supplementary Table 1). Next, beta diversity assessed by Principal Co-ordinate Analysis (PCoA) showed significantly distinct clusters between men and women along the PC1 axis as depicted in Figure 1C. Overall, there were sex-based differences in gut microbial diversity in terms of abundance as measured by alpha diversity and composition as indicated by beta diversity.

**Figure 2:**
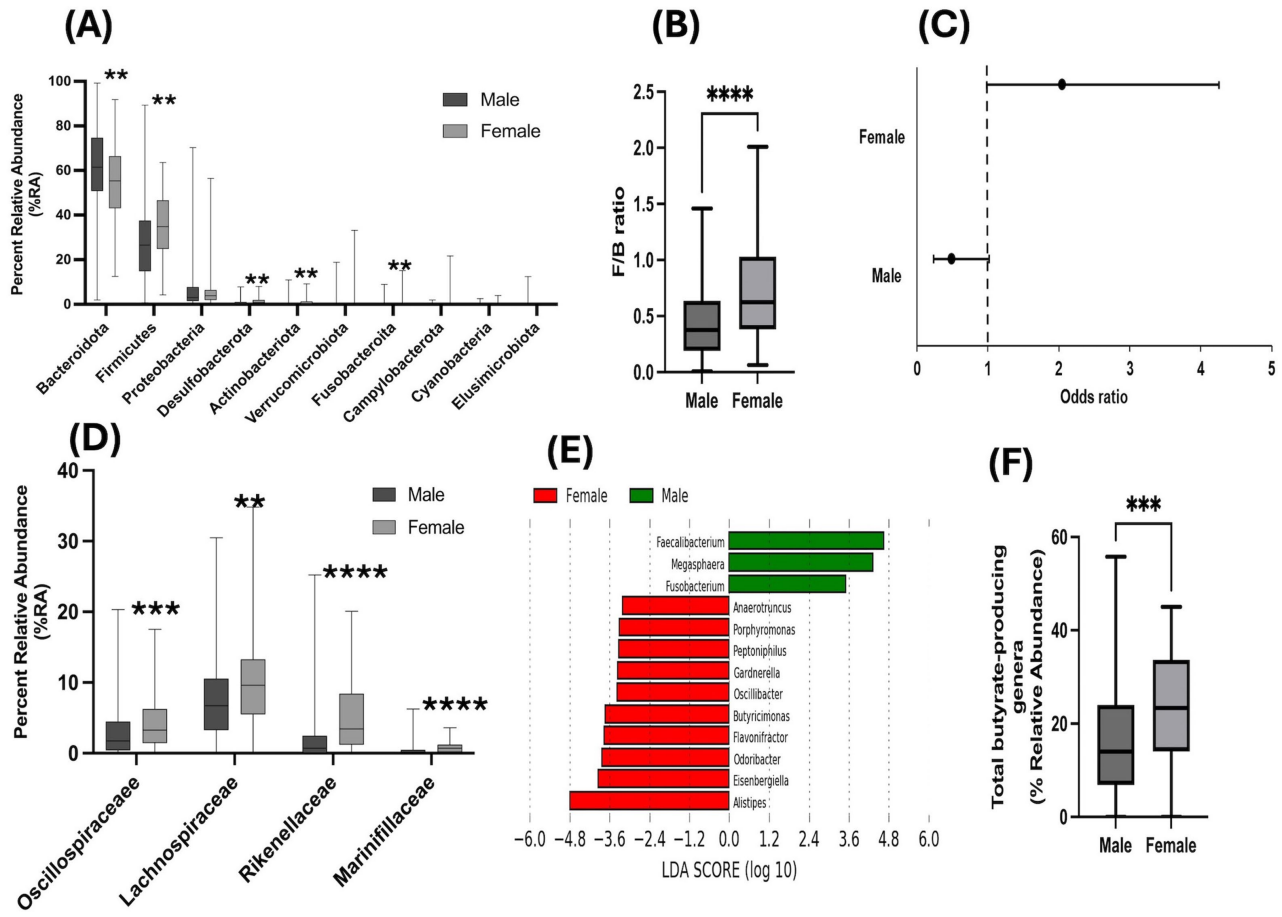
Sex-based differences in gut microbiome compositional changes from phyla to genera levels of taxonomy: Bar graphs depicting unadjusted Mann–Whitney *U*-test analysis for **(A)** %RA of bacteria at the phyla level; **(B)** ratio of %RA of Firmicutes to Bacteroidota phyla (F/B ratio); **(C)** Odds of having F/B ratio >1 in women compared to men; figure represents Ors and 95% Cis; graphs showing **(D)** higher %RA of butyrate-producing families; **(E)** LEfSe analysis depicting enrichment of higher number of butyrate-producing genera and **(F)** higher %RA of total butyrate-producing genera in women compared to men. Statistical analysis shown as **p < 0.01, ***p < 0.001, ****p < 0.0001.

Further, examination of the top 10 most abundant phyla demonstrated sex-based differences in their distribution, with women depicting significantly higher %RA of *Firmicutes*, *Desulfobacteriota*, *Actinobacteriota*, *Fusobacteriota* and lower %RA of *Bacteriodota* compared to men (Figure 2A). Importantly, women showed significantly higher F/B ratio compared to their male counterparts (Figure 2B). Further, logistic regression analysis was employed to evaluate the effect of sex on F/B ratio. The data revealed that women had 104% higher odds of having F/B ratio greater than 1 compared to men (Figure 2C, *P*-value = 0.055, model accuracy = 0.82). Additionally, taxonomic analysis of bacterial families demonstrated that in comparison to men, women had significantly greater %RA of *Marinifilacea, Rikenellacea, Bacteriodacea, Leptotrichiacea, Barnesiellacea, Oscillospiracea, Tannerallacea, Desulfovibrionacea, Lachnospiracea* and *Victivallacea* and significantly lesser %RA of *Prevotellacea, Selenomonadacea, Succinivibrionacea* (Supplementary Table 2). This further corroborated the sex-based differences in bacterial diversity and gut dysbiosis observed in this study population.

### Sex-based differential abundance of beneficial butyrate-producing bacteria is a hallmark feature of gut dysbiosis in PWH with a history of heavy alcohol drinking

Since both heavy alcohol drinking and HIV-1 infection have been independently linked to loss of beneficial butyrate-producing bacteria (Dillon et al., 2017; Johnson and Byrareddy, 2022; Du et al., 2022), we examined the sex-based distribution of butyrate-producing families in our study cohort. Our analysis showed that women harbored significantly greater %RA of butyrate-producing families *Oscillospiracea* and *Lachnospiracea* that belong to the *Firmicutes* phylum and *Marinifilacea* and *Rikenellacea* that belong to the *Bacteriodota* phylum (Figure 2D).

For further detailing of the microbiome at the genus level of taxonomy, we employed an in-house database of butyrate-producing bacterial genera (Singhal et al., 2021; Aakarsha, 2025) and performed a LEfSe analysis. The data demonstrated a greater number of butyrate-producing genera were enriched in women compared to men (Figure 2E). Moreover, %RA of total butyrate-producing genera was also observed to be significantly greater in women (Figure 2F; *p* < 0.001). Importantly, when adjusted for covariates considered in this study, women continued to harbor significantly greater %RA of total butyrate-producing genera compared to men (Supplementary Table 1). Taken together, these data demonstrated that there are sex-based differences in butyrate-producing bacteria in this study cohort.

**Figure 3:**
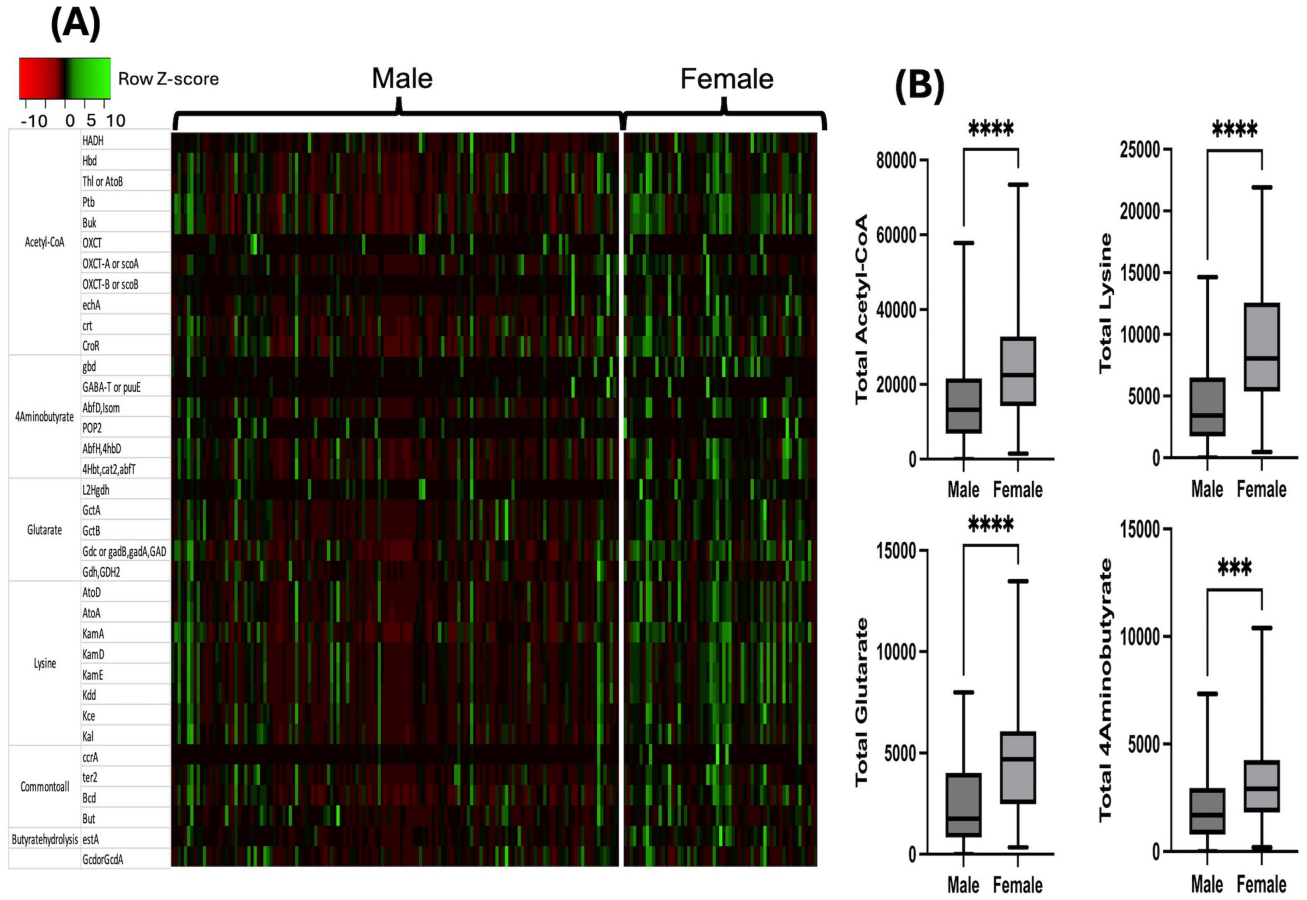
Sex-based differences in butyrate synthesizing bacterial genes and pathways: inferred metagenomics analysis using PICRUSt2. **(A)** Heatmap demonstrating the differences in predictive functional profile. Red color signifies low expression or absent and green color signifies high expression or present and **(B)** Bar graphs depicting unadjusted Mann-Whiteny *U*-test comparison of relativized gene expression from the four major butyrate synthesizing pathways. Statistical analysis shown as *p < 0.05; **p < 0.01; ***p < 0.001; ****p < 0.0001. HADH – 3-hydroxyacyl-CoA dehydrogenase; Hbd – *β*-hydroxybutyryl-CoA dehydrogenase; Thl or AtoB – Thiolase; Ptb – Phosphate butyryltransferase; Buk – butyrate kinase; OXCT – 3-oxoacid CoA transferase; OXCT-A or scoA – 3-oxoacid CoA-transferase subunit A; OXCT-B or scoB – 3-oxoacid CoA-transferase subunit B; echA, crt, CroR – crotonase; gbd – 4-hydroxybutyratedehydrogenase; GABA-T or puuE – 4-aminobutyrate transaminase; AbfD or Isom – 4-hydroxybutyryl-CoA dehydratase; POP2–4-aminobutyrate-pyruvate transaminase; AbfH, 4hbD – 4-hydroxybutyrate dehydrogenase; 4Hbt, cat2, abfT – butyryl-CoA:4-hydroxybutyrate CoA transferase; L2Hgdh – 2-hydroxyglutarate dehydrogenase; GctA – glutaconate CoA transferase (*α*); GctB – glutaconate CoA transferase (β); Gdc or gadB, gadA, GAD – Glutamate decarboxylase; Gdh, GHD2 – Glutamate dehydrogenase; AtoD – butyryl-CoA:acetoacetate CoA transferase (α subunit); AtoA – butyryl-CoA:acetoacetate coA transferase (β subunit); KamA – lysine-2,3-aminomutase; KamD – β-lysine-5,6-aminomutase α; KamE – β-lysine-5,6-aminomutase β; Kdd – 3,5-diaminohexanoate dehydrogenase; Kce – 3-keto-5-aminohexanoate cleavage enzyme; Kal – 3-aminobutyryl-CoA ammonia lyase; ccrA – crotonyl-CoA reductase; ter2 – enoyl-acyl-carrier protein reductase; Bcd – butyryl-CoA dehydrogenase (including electron transfer protein α, β subunits); But – butyryl-CoA:acetate CoA transferase; estA – putative tributyrin esterase; Gcd or GcdA – glutaconyl-CoA decarboxylase (α, β subunits).

Following metataxonomic analysis, inferred metagenomics analysis of the sex-based differences in the butyrate synthesizing genes was performed by PICRUSt2, employing the butyrate genes inventory (Singhal et al., 2021; Douglas et al., 2020; Vital et al., 2017; Vital et al., 2014). Corresponding to sex-based differences in total butyrate-producing bacteria, the data demonstrated that women have significantly greater abundance of genes involved in butyrate synthesis through four major pathways namely Acetyl-CoA, Glutarate, 4-Aminobutyrate and Lysine (Figures 3A,B).

### Sex-based differences in percent relative abundance of *Prevotellaceae* in PWH with a history of hazardous alcohol consumption

**Figure 4:**
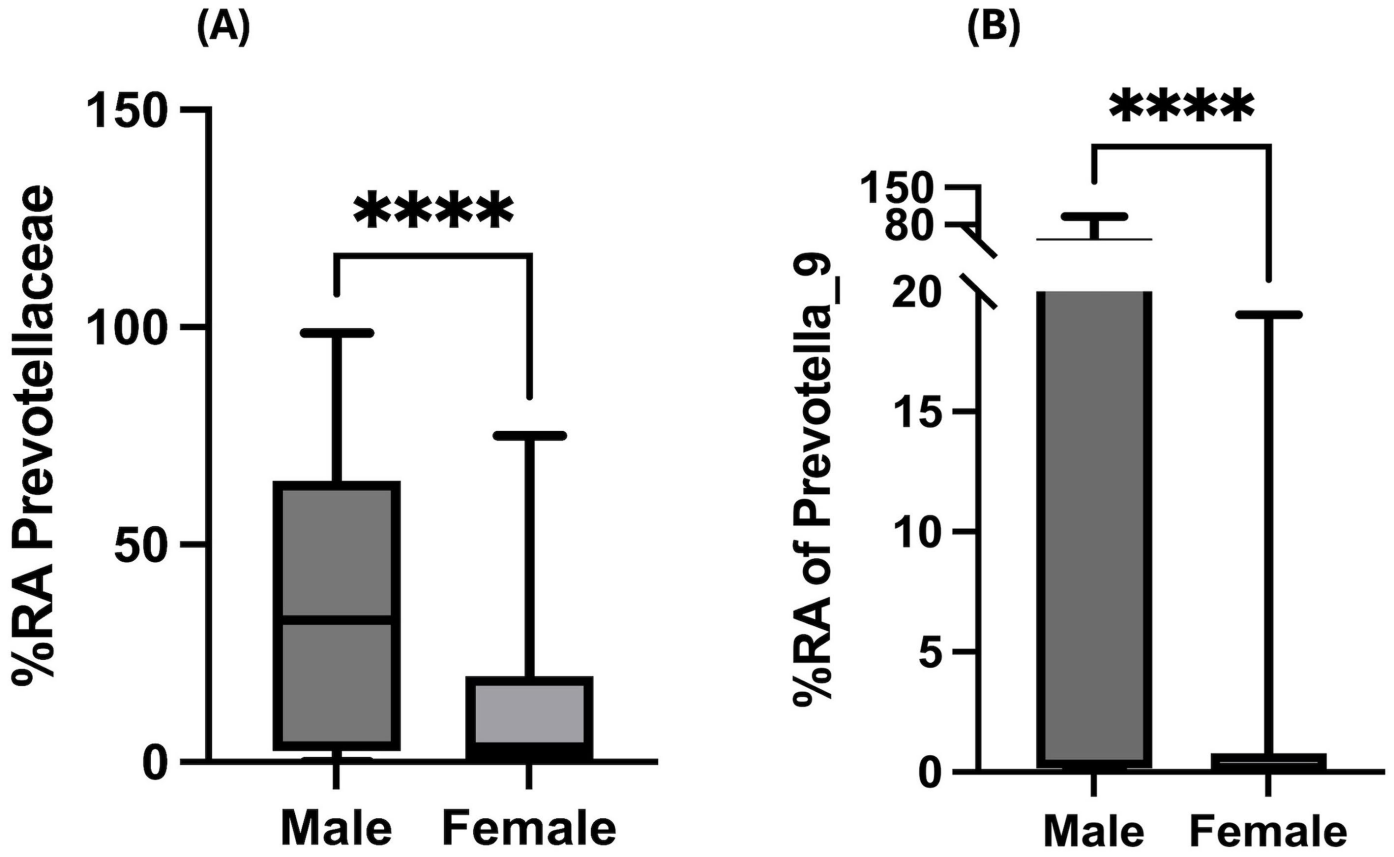
Sex-based differences in gut microbial pathobionts: Bar graphs depicting unadjusted Mann–Whitney U-test comparison of sex-based differences in **(A)** %RA of Prevotellaceae family and **(B)** Prevotella_9 genus among PWH with a history of hazardous alcohol drinking. Statistical analysis shown as ****p < 0.0001.

In the context of HIV infection induced gut microbial dysbiosis, in addition to loss of beneficial butyrate-producing bacteria (Dillon et al., 2017), higher abundance of *Prevotellaceae* has also been reported (Lozupone et al., 2013). However, sex-based differences in the relative abundance of *Prevotellaceae* family in PWH warrants examination. In our study population, women depicted significantly lesser %RA of *Prevotellaceae* in comparison to their male counterparts (Figure 4A; *p* < 0.0001). Importantly, this observation remained statistically significant even after adjusting for confounding factors considered in this study (Supplementary Table 1). Among *Prevotellaceae* family, *Prevotella_9* was the most predominant genus that showed significant difference between men and women, with women depicting significantly lesser %RA (Figure 4B; *p* < 0.0001).

Overall, our observations suggest that although the cohort comprises of PWH with a history of heavy alcohol drinking, there are salient features that characterize sex-based differences in the structure and function of their gut microbiome.

## Discussion

HIV-1 infection continues to be a serious public health challenge (Nikolopoulos and Tsantes, 2022; Seyler et al., 2018; Bekker et al., 2023). Among PWH, hazardous alcohol drinking is prevalent and is known to exacerbate HIV pathogenesis and associated comorbidities (Crane et al., 2017; Justice et al., 2016). Additionally, sex is an important determinant of health that can influence the pathophysiology of HIV infection (Scully, 2018; Moran et al., 2022). In this regard, Center for Disease Control and Prevention (CDC) reported that prevalence of newly diagnosed cases of HIV-1 infection is four times higher in men than women (Prevention CfDCa, 2023). Moreover, excessive alcohol consumption is also observed at higher rates in men compared to women (Kezer et al., 2021; Wilsnack et al., 2009). However, studies examining the combinatorial effects of HIV-1 infection and heavy alcohol consumption among men and women are scarce (Duko et al., 2019). The present study investigates the sex-based differences gut microbiome changes in PWH who have a history of hazardous alcohol drinking.

A growing body of evidence has shown that gut microbiota plays an important role in disease pathogenesis. Earlier work has demonstrated that HIV-1 infection and heavy alcohol drinking can independently lead to gut microbial dysbiosis, microbial translocation and systemic inflammatory responses (Mutlu et al., 2012; Calleja-Conde et al., 2021; Mutlu et al., 2014; Nowak et al., 2015). With regards to combinatorial effects of heavy alcohol drinking and HIV-1 infection, current literature merely surmises that they have an additive detrimental effect on gut microbial dysbiosis (Yan et al., 2021; Molina et al., 2018). Additionally, evaluation of gut microbiome changes in healthy individuals showed that there are inherent sex-based differences in bacterial composition (Kim et al., 2020; Mueller et al., 2006; Ding and Schloss, 2014; Koliada et al., 2021; Takagi et al., 2019). Hence, research characterizing the sex-based alterations in the structure and function of the gut microbiome as a consequence of heavy alcohol drinking in PWH is needed. In this regard, the present study showed significant differences in gut microbial diversity and composition with higher percent relative abundances of butyrate producing beneficial bacteria and lower percent relative abundance of pathobionts in women compared to men.

In our population, a significant difference in gut microbial diversity was observed between men and women. Specifically, alpha diversity measured as OTUs and Shannon Index was significantly greater in women than men. Sex-based differences in the microbial alpha diversity have also been reported in cohort of individuals without HIV-1 infection and/or heavy alcohol drinking (Sinha et al., 2019); however, the observed degree of difference in the alpha diversity is significantly lower compared to our study population. Hence, the present data implicate that both HIV-1 infection and hazardous alcohol drinking have a negative impact on the gut microbiome that further exacerbates the inherent sex-based differences in the gut microbial diversity. Moreover, at the phyla level, in the present study, percent relative abundances (%RA) of *Firmicutes*, *Desulfobacterota*, and *Fisobacteriota* phyla were significantly higher in women while that of *Bacteroidota* phylum was lower in women compared to men. Notably, these sex-based differences at the phyla level were also observed in a population involving healthy individuals from a country in the European continent namely Ukraine, albeit at a significantly lesser degree. Furthermore, the population from Ukraine demonstrated 31% higher odds of women having F/B ratio greater than 1 compared to men (Koliada et al., 2021). Similar calculations involving patients from our study cohort demonstrated that women have twice the odds of having F/B ratio greater than 1 compared to men. Since F/B ratio is considered as a marker of gut microbial dysbiosis (Stojanov et al., 2020), our data indicates that there is a pronounced effect of the combination of hazardous alcohol drinking coupled with HIV-1 infection.

The beneficial contributions of butyrate-producing bacterial communities to the health and maintenance of the human gut have been discussed in detail (Singh et al., 2022). Therefore, we utilized an in-house well-substantiated repository of butyrate-producing genera to delineate sex-based differences in the %RA of individual as well as total butyrate-producing genera in our cohort (Singhal et al., 2021). Our analyses demonstrated significant sex-based differences with higher abundance of several individual and total butyrate-producing genera in women compared to men. Further, the PICRUSt2 analysis indicated that in comparison to men, women depicted significantly higher relativized expression of bacterial genes belonging to all four major pathways involved in butyrate synthesis namely, acetyl-CoA, 4-Aminobutyrate, glutarate and lysine. In addition to sex-based differences in putative bacterial gene expression, our PICRUSt2 analysis also revealed that among the 4 butyrate synthesizing pathways, acetyl-CoA was the most abundantly utilized pathway by the gut bacteria to produce butyrate. Since men in this cohort drink more alcohol (number of drinks per week, Table 1) than women, this data is in line with our earlier preclinical study where mice exposed to chronic alcohol had a significant reduction in butyrate-producing bacteria, specifically the abundance of acetyl-CoA utilizing bacteria, suggesting the role of alcohol in altering the butyrate community in men (Singhal et al., 2021).

Besides loss of beneficial bacteria, HIV-associated gut microbial dysbiosis can also be marked by an increase in pathogenic bacteria. A prominent HIV-associated microbiome alteration observed by several groups is an increase in the genus Prevotella (Lozupone et al., 2013; Mutlu et al., 2014; Dillon et al., 2014; Vázquez-Castellanos et al., 2015). Importantly, along with HIV status, influence of sexual orientation has been observed to play a major role in the enrichment of Prevotella in the gut microbiome. In this regard, studies on US-based populations have shown that fecal and rectal microbiomes from HIV-infected men having sex with men (MSM) are enriched in Prevotella. It is important to note that Prevotella-rich microbiome was present in MSM regardless of their HIV infection status (Neff et al., 2018; Noguera-Julian et al., 2016). In contrast, men from our Russian study cohort who are predominantly men having sex with women (MSW; ~90%, Table 1) depicted a Prevotella-rich microbiome (~36.25%RA). Further, with regards to sex-based differences in the gut microbiome, men had a significantly greater increase in %RA of *Prevotellaceae* than women even after adjustment for the HIV viral load and alcohol use. Hence, the enrichment of *Prevotellaceae* observed in our MSW population was not influenced by HIV viral load, alcohol use and sexual orientation (MSM), and needs further investigation. Moreover, our study also suggests that there is a need to consider diverse populations and geographical contexts when evaluating HIV-associated microbiome changes.

We acknowledge that our study has certain limitations. Only a cross-sectional investigation was performed to establish a conceptual framework to determine the combinatorial effect of heavy alcohol consumption and HIV-1 infection on gut microbiome changes that would be clinically relevant for disease pathogenesis. Since the incidence of HIV-1 infection is predominantly seen in men compared to women, our cohort with small sample size also reflects a similar distribution where the cohort consists of approximately 3 times higher proportion of male participants (*n* = 143) than female (*n* = 59). Hence, further studies using a larger study cohort in a longitudinal setting will be required to validate the initial findings made in this study and support a potential causal role for sex-based imbalance in gut microbiome composition with decreased butyrate-producing bacteria and increased pathobionts (e.g., Prevotellaceae) in HIV pathogenesis. Considering the qualitative nature of some of the measures, e.g., alcohol consumption assessed by self-report, the data analysis has been adjusted for clinically relevant cofactors such as log10 of HIV-viral load, CD4-count, opioid use and number of drinks consumed per week. Interestingly, results remain significantly different in the context of sex, when adjusted for confounding factors. Moreover, our study lacks the comparative assessment with healthy controls and/or groups consisting of only PWH or only heavy alcohol drinkers, which would allow further delineation of sex-specific gut microbiome differences among PWH with a history of heavy alcohol consumption. However, several studies have documented the microbiome changes independently in the context of HIV infection and heavy alcohol consumption. In addition, it is difficult to know whether the findings from the study are specific to the geographical region (Russia) or can represent PWH who are heavy alcohol drinkers elsewhere. Therefore, these findings should be replicated in cross-cohort collaborations so that we can better understand the sex-dependent microbiome changes and comment further on the generalizability of these findings. Lastly, we recognize that the inferred metagenomics results presented through PICRUSt2 warrants further validation with confirmatory assessments such as gene expression through qPCR. Since our focus was the only on the delineation of differences in the structural features of the gut microbiome in this study cohort, future studies will delve further into characterization of the functional consequences such as plasma butyrate levels that mirror the sex-based compositional differences of the gut microbiome.

In summary, the present work identified significant sex-based differences in the gut microbiome of PWH who have a history of heavy alcohol consumption. Specifically, these differences were observed in the relative abundances of beneficial butyrate-producing bacteria and the potential pathogenic Prevotella. Despite successful viral suppression in PWH through ART, persistent gut microbial dysbiosis has been observed to significantly contribute to chronic inflammation and various comorbidities (Pinto-Cardoso et al., 2025; Ishizaka et al., 2024). This highlights the clinical need of addressing gut dysbiosis to improve the long-term health outcomes for PWH. Importantly, these data have the potential to support the future development and clinical trials of evidence-based probiotics to address the specific gut microbial changes observed in men and women, to improve gut health and mitigate the associated comorbidities in this vulnerable population.

## Data Availability

The data presented in this study has been deposited to the NCBI repository (https://www.ncbi.nlm.nih.gov/) under BioProject ID PRJNA1353104 and BioSample ID: SAMN52927815.
