# Supplementary material for: Sex differences in beneficial and pathogenic bacteria in People With HIV (PWH) with a history of heavy alcohol drinking
Source: Front Microbiol. 2025 Nov 21;16:1632949. doi: 10.3389/fmicb.2025.1632949 (PMC12679886; doi:10.3389/fmicb.2025.1632949)
Supplement: Supplementary file 1 [file Table_1.docx]

**Supplementary Tables**

**Supplementary Table 1: Summary of multivariate linear regression model (unadjusted and adjusted for confounding factors considered in this study).**

***The multivariate regression model has been adjusted for covariates including age, log10 of HIV viral load, current ART usage, CD4 count, opioid usage over 30 days and number of drinks consumed per week over 30 days.**

|  | **Unadjusted** | | **Adjusted*** | |
| --- | --- | --- | --- | --- |
|  | **Female vs. Male** | | **Female vs. Male** | |
|  | **Ration of means (95% CI)** | **p-value** | **Ration of means (95% CI)** | **p-value** |
| **Natural log of F/B ratio** | 1.56 (1.13, 2.15) | 0.007 | 1.55 (1.12, 2.15) | 0.009 |
|  | **Difference in means (95% CI)** | **p-value** | **Difference in means (95% CI)** | **p-value** |
| **Operational Taxonomic Units (OTUs)** | 13.5 (3.4, 23.7) | 0.009 | 14.6 (4.4, 24.8) | 0.005 |
| **Shannon Index** | 0.51 (0.29, 0.73) | <0.001 | 0.53 (0.31, 0.75) | <0.001 |
| **%RA of Prevotellaceae** | -22.4 (-31.0, -13.7) | <0.001 | -22.8 (-31.5, -14.0) | <0.001 |
| **%RA of total butyate-producing genera** | 6.9 (3.2, 10.5) | <0.001 | 7.2 (3.6, 10.9) | <0.001 |

**Supplementary Table 2: Sex-based differences in top 15 families of bacterial taxonomy. Mann-Whitney U tests were performed; multiple tests were corrected for using Benjamani Hochberg method.**

| **Taxa** | **Test** | **Statistic** | **P-value** | **FDR-Adj. P** | **Mean rank of Male** | **Mean rank of Female** |
| --- | --- | --- | --- | --- | --- | --- |
| Marinifilaceae | Mann-Whitney | 2441 | 0.000001 | 0.000016 | 89.07 | 131.6 |
| Rikenellaceae | Mann-Whitney | 2468 | 0.000002 | 0.000016 | 89.26 | 131.2 |
| Prevotellaceae | Mann-Whitney | 2539 | 0.000006 | 0.00003 | 113.2 | 73.03 |
| Bacteroidaceae | Mann-Whitney | 2629 | 0.000019 | 0.000073 | 90.38 | 128.4 |
| Leptotrichiaceae | Mann-Whitney | 3718 | 0.000139 | 0.000418 | 98 | 110 |
| Barnesiellaceae | Mann-Whitney | 2942 | 0.000275 | 0.000687 | 92.57 | 123.1 |
| Oscillospiraceae | Mann-Whitney | 2956 | 0.000753 | 0.001614 | 92.67 | 122.9 |
| Tannerellaceae | Mann-Whitney | 2992 | 0.001047 | 0.001963 | 92.92 | 122.3 |
| Selenomonadaceae | Mann-Whitney | 3253 | 0.001726 | 0.002876 | 108.3 | 85.13 |
| Desulfovibrionaceae | Mann-Whitney | 3100 | 0.002745 | 0.004117 | 93.68 | 120.5 |
| Succinivibrionaceae | Mann-Whitney | 3658 | 0.003286 | 0.004481 | 105.4 | 92 |
| Lachnospiraceae | Mann-Whitney | 3141 | 0.004158 | 0.005198 | 93.97 | 119.8 |
| Victivallaceae | Mann-Whitney | 3531 | 0.014365 | 0.016575 | 96.69 | 113.2 |
| Erysipelotrichaceae | Mann-Whitney | 3393 | 0.024758 | 0.025272 | 95.73 | 115.5 |
| Bifidobacteriaceae | Mann-Whitney | 3415 | 0.025272 | 0.025272 | 95.88 | 115.1 |
